# Molecular Pathogenesis of Fibrosis, Thrombosis and Surfactant Dysfunction in the Lungs of Severe COVID-19 Patients

**DOI:** 10.3390/biom12121845

**Published:** 2022-12-10

**Authors:** Adrian Krygier, Dagmara Szmajda-Krygier, Rafał Świechowski, Jacek Pietrzak, Agnieszka Wosiak, Damian Wodziński, Ewa Balcerczak

**Affiliations:** Laboratory of Molecular Diagnostics and Pharmacogenomics, Department of Pharmaceutical Biochemistry and Molecular Diagnostics, Medical University of Lodz, Muszynskiego 1, 90-151 Lodz, Poland

**Keywords:** SARS-CoV-2, COVID-19, pulmonary fibrosis, cytokine storm, ARDS, pulmonary surfactant, hypercoagulability

## Abstract

The global scope and scale of the SARS-CoV-2 pandemic led to huge amounts of important data from clinical observations and experimental analyses being collected, in particular, regarding the long-term impact of COVID-19 on lung tissue. Visible changes in lung tissue mainly relate to the destruction of the alveolar architecture, dense cellularity, and pulmonary fibrosis with myofibroblast proliferation and collagen deposition. These changes are the result of infection, mainly with virus variants from the first pandemic waves (Alpha to Delta). In addition, proper regulation of immune responses to pathogenic viral stimuli is critical for the control of and recovery from tissue/organ damage, including in the lungs. We can distinguish three main processes in the lungs during SARS-CoV-2 infection: damage or deficiency of the pulmonary surfactant, coagulation processes, and fibrosis. Understanding the molecular basis of these processes is extremely important in the context of elucidating all pathologies occurring after virus entry. In the present review, data on the abovementioned three biochemical processes that lead to pathological changes are gathered together and discussed. Systematization of the knowledge is necessary to explore the three key pathways in lung tissue after SARS-CoV-2 virus infection as a result of a prolonged and intense inflammatory process in the context of pulmonary fibrosis, hemostatic disorders, and disturbances in the structure and/or metabolism of the surfactant. Despite the fact that the new Omicron variant does not affect the lungs as much as the previous variants, we cannot ignore the fact that other new mutations and emerging variants will not cause serious damage to the lung tissue. In the future, this review will be helpful to stratify the risk of serious complications in patients, to improve COVID-19 treatment outcomes, and to select those who may develop complications before clinical manifestation.

## 1. Introduction

The pandemic caused by SARS-CoV-2 has affected more than 200 million people to date. According to the WHO (World Health Organization) and the CRC (Coronavirus Resource Center at Johns Hopkins University), more than 4 million deaths have been recorded and 180 million people have recovered. The scale of the pandemic is a central reason to select and order the data from available publications written on the basis of clinical observations and experimental analysis in order to support people who have recovered. In addition, the long-term effects of COVID-19 on the lungs and further biochemical and physiological processes in other tissues remain unclear [1,2,3]. 

SARS-CoV-2 virus infection, which was observed during the first waves of the pandemic, is not only characterized by multiple organ involvement in the acute phase of infection but also may result in a high incidence of post-acute sequelae of SARS-CoV-2 virus infection (PASC) [4,5,6]. PASC is becoming an important concern in SARS-CoV-2-infected people, with approximately 10–30% of COVID-19 survivors experiencing effects after 3-6 weeks of acute onset. The most frequent clinical symptoms of PASC are fatigue, dyspnea, fear, and other mental disorders, including depression. Moreover, in many patients, after SARS-CoV-2 virus infection, visible changes in the lung tissue are found. These changes mainly concern alveolar architectural destruction, dense cellularity, and pulmonary fibrosis (PF) with myofibroblast proliferation and collagen deposition. However, it is worth mentioning that many biochemical and pathological abnormalities have been reported in the renal, cardiovascular, and coagulation systems that differ from those occurring in disseminated intervascular coagulation syndrome (DIC) as a consequence of immunological response [4]. Proper regulation of immune responses to pathogenic viral stimuli is crucial for the control of and recovery from tissue/organ injury. The lungs are the main reservoir of the virus and this is where the greatest damage occurs. Therefore, in this study, we mainly focus on the abnormalities in the activation of this organ and the recruitment of inflammatory cells leading to the release of inflammatory mediators, which correlate with the disease characteristics, such as loss of lung function, airway hyperresponsiveness and obstruction, edema, mucus hypersecretion, and remodeling of lung tissue [7]. The response from the immunological system could also be triggered by pulmonary surfactant (PS), a protein that plays a crucial role in maintaining of the surface tension, which is necessary for efficient gaseous exchange at the air–liquid interface of the alveoli [8]. 

In COVID-19-related lung disease, there are also two distinct phenotypes induced by inflammation and other processes connected with fibrosis. The first phenotype is COVID-19-related acute respiratory distress syndrome (ARDS), which exhibits a classical histopathological pattern of fibrotic diffuse alveolar damage (DAD) in exudative and organizing phases, with or without pulmonary artery thrombosis in different stages of development, as was confirmed in postmortem analyses [9]. The second phenotype is post-COVID pulmonary fibrosis (PCPF) [10]. The pathogenic progression of pulmonary fibrosis post-COVID-19 is multifactorial and considered to be due to the abnormal healing process of the injured lung parenchyma. The clinical characteristics, risk factors, biomarkers, and pathophysiology are different for both phenotypes. Moreover, the prognosis in these two phenotypes and the optimal treatment, which includes antifibrotic drugs, is very difficult and must include all coexisting abnormalities [11,12,13]. 

**Figure 1:**
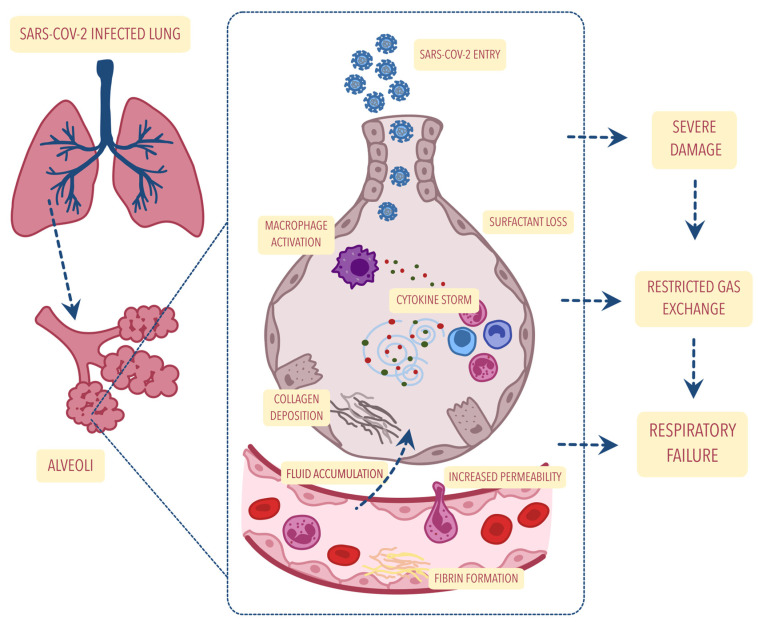
Alveolar changes in the course of COVID-19 leading to severe damage. This then leads to limited gas exchange resulting in respiratory failure. The figure was created using the Vectornator application (version 4.11.4 for iOS, accessed on 25 October 2022).

Moreover, patients infected with the SARS-CoV-2 virus frequently develop bilateral pneumonia with accompanying damage to the cells that produce the lung surfactant. During early period of the COVID-19 pandemic, reports stated that up to 40% of infected patients developed viral pneumonia and that 20% of these individuals developed severe ARDS [14,15]. At first, COVID-19 causes diffuse alveolar damage analogous to the pathology observed in other forms of viral pneumonia, followed by distinct angiocentric features, e.g., severe endothelial damage, microangiopathy, and obstruction of the alveolar capillaries. After that, a depletion in lung surfactant activity and a reduction in the content and structure of active large surfactant aggregates are observed as a result of various mechanisms, e.g., the interaction between surfactant and inflammatory edema and the inhibition of surfactant aggregates formation [7]. During CARDS, surfactant deficiency may also occur as a result of virus-induced type II pneumocytes lysis with the associated formation of a hyaline membrane [6], radiographic evidence of ground-glass opacities, bilateral infiltrates, a reduction in lung compliance, and refractory hypoxemia [8]. Figure 1 summarizes the alveolar changes observed in the course of COVID-19 leading to respiratory failure.

However, it is worth mentioning that currently the SARS-CoV-2 Omicron variant has spread rapidly worldwide and has practically replaced other virus variants. This new variant was first reported in South Africa on 24 November 2021. Available data suggest that the Omicron variant is less likely to cause severe disease than other variants. It has also been observed that the Omicron variant is rarely a cause of pneumonia due to its low replication capacity in the lung parenchyma—with the incidence of pneumonia decreasing from 77% to 34.2% [16,17,18]. Moreover, the odds of hospitalization and of severe disease in patients with Omicron infection are half than those attributed to patients infected with the Delta and preceding variants [19]. On the other hand, another study reported that the SARS-CoV-2 Omicron variant can probably cause inflammation along the bronchi, which results in bronchial pneumonia [16]. What is more, the Omicron variant is known to differ from earlier variants, such as Alpha and Delta, and is characterized by rapid replication in bronchial epithelial cells [16,19,20]. It cannot be overlooked that this variant is a heavily mutated virus, and it has been designated as a variant of concern by the World Health Organization (WHO) [17,21]. These variants are the result of spontaneous mutations in the viral RNA resulting from replication errors in the host cell. The most common mutations observed in the Omicron variant are associated with the gene encoding the S protein and include A67V, del69-70, T95I, G142D, del143-145, Y145D, del211, L212I, and ins214EPE [22]. Despite the fact that the new Omicron variant does not affect the lungs as much as the previous ones, we cannot ignore the fact that other new mutations and emerging variants will not cause serious damage to the lung tissue. There is still a lack of studies evaluating lung imaging results for Omicron infection compared to other variants.

The systematization of our knowledge is necessary to explore the three key changes taking place in the lung tissue after SARS-CoV-2 virus infection (from the Alpha to Delta variants) as a result of a long-lasting and intense inflammatory process in the context of pulmonary fibrosis, hemostatic disorders, and disturbances in the structure and/or metabolism of the surfactant. 

Although the above-mentioned disorders mainly occur among patients with a severe course of disease, accounting for about 15–20% of cases, the global scale of the early phase of the pandemic translated into a huge number of affected people. In the present work, data on the abovementioned three biochemical processes that lead to pathological changes are gathered together and discussed. 

## 2. Pulmonary Surfactant during SARS-CoV2 Virus Infection

Pulmonary surfactant is an important component of the normal lung tissue. PS lines the pulmonary alveolar surface and plays a crucial role in facilitating inflation and deflation during breathing [23]. Furthermore, PS is involved in numerous nonmechanical biological processes, such as host defense and lipid homeostasis, and most chronic respiratory pathologies are related to substantial changes in pulmonary surfactant homeostasis, as demonstrated in various preclinical and clinical studies [24]. 

Although many studies have demonstrated that surfactant is responsible for homeostatic processes in the lungs, i.e., gas exchange and host defense, it is primarily known for lowering alveolar surface tension. The surfactant is essential for proper lung function as it reduces surface tension, which is facilitated by the major phospholipid component—phosphatidylcholine (PC)—especially in the form of unsaturated dipalmitoylphosphatidinecholine (DPPC). This enables efficient lung inflation and deflation while preventing the small airways from collapsing. Moreover, the effectiveness of this gas exchange can be significantly influenced by changes in the amount, composition, and structure of the surfactant, since the exchange of oxygen and carbon dioxide between the air space and the lung parenchyma takes place through the surfactant layer at the air–liquid interface [24,25,26,27,28,29]. 

The alveolar epithelium is composed of alveolar type I (ATI) cells and alveolar type II (ATII) cells. ATII cells make up approximately 5–7% of the alveolar epithelial cells and are small cuboid-shaped cells with an area of approximately 250 µm^2^ [30,31,32]. The ATII cells serve multiple roles, one of them being surfactant synthesis. The exocytosis of surfactant complexes in the form of lamellar bodies and the majority of their reuptake and recycling is mediated by these cells. When lamellar bodies are secreted and combined with surfactant proteins in the aqueous layer of the hypophase, cylindrical myelin forms are created, which serve as a resource of the phospholipids that are transferred into the surfactant monolayer at the alveolar air–liquid interface [33]. ATII cells can also secrete cytokines, growth factors, and endogenous antimicrobial peptides, which affect the immune system response [30,31,32,33,34]. 

The pulmonary surfactant mainly consists of lipids and proteins. Lipids constitute up to 90% of the surfactant composition and include phospholipids (mainly DPPC) and other neutral lipids, such as cholesterol. The lipids contained in the surfactant are primarily responsible for reducing the air–fluid surface tension inside the alveoli and preventing atelectasis. The remaining 10% of the composition is made of proteins. The main proteins that make up the pulmonary surfactant are SP-A, SP-B, SP-C, and SP-D. The SP-A and SP-D proteins, commonly referred to as collectins, are hydrophilic and are crucial for sustaining lung immunity by eliminating viruses and bacteria. The surfactant proteins SP-B and SP-C are hydrophobic and are essential in reducing the surface tension at the respiratory air–liquid interface [35,36,37]. Various novel surfactant-related proteins (SFTAs) that exhibit similar properties to standard SP have been recently described, namely, SFTA2, which is a hydrophilic protein and exhibits similar functions to SP-A and SP-D. Additionally, the SFTA3, an amphiphilic protein, improves phagocytosis in macrophage cell lines. Surfactant phospholipids are captured by ATII cells or alveolar macrophages and are taken up for recycling or degradation over time or following direct damage, i.e., exposure to inhaled oxidants/toxins [38,39,40,41].

### 2.1. Etiopathogenesis of PS Damages in COVID-19 Patients 

The available scientific data suggest that pulmonary surfactant plays an important role in COVID-19 infection and affects its severity and course. In the case of COVID-19 lung infection, SARS-CoV-2 damages type II alveolar cells, reducing the synthesis of pulmonary surfactant and its secretion into the alveolar space, which ultimately leads to lung failure [23,42,43]. 

The entry of the SARS-CoV-2 virus through lungs is mediated by binding viral Spike protein (S-protein) with an angiotensin-converting enzyme 2 (ACE2) receptor. By linking to ACE2 receptor, the SARS-CoV-2 virus is thought to destroy the ATII cells, which results in a decrease in surfactant synthesis and its secretion into the alveolar space, and it has a consequence in reduced protein S (pS) production. The reduction in surfactant synthesis causes atelectasis and decreases pulmonary compliance [44,45]. 

Although the exact percentage data on the frequency of dysfunction of the surfactant itself have not been reported thus far, the recent systematic review prepared by Torres-Castro et al. stated that in the context of impaired diffusion and restrictive physiology in lung function, a reduced diffusive capacity was observed in 39%, a restrictive ventilation pattern was observed in 15%, and an obstructive ventilation pattern was observed in 7% of COVID-19 infection survivors after first waves of the pandemic [15,46].

COVID-19 patients appear to present clinical symptoms much like those observed in neonatal respiratory distress syndrome (NRDS) [30,47,48]. The primary cause of NRDS is pulmonary surfactant deficiency, which has consequences in lung atelectasis, and ARDS–SARS-CoV-2-infected patients also exhibit these symptoms. Moreover, in patients infected with SARS-CoV-2, impaired pulmonary surfactant function disturbs the air–liquid interphase. Alveolar cell loss results in a decrease in blood oxygen levels, contributes to pulmonary fibrosis, hinders regeneration, and causes edema, all of which can lead to respiratory system failure [23,35,49]. The specific sequence of changes in the lungs of COVID-19 patients evaluated by transmission electron microscopy includes epithelial cell damage at the outset, which is further accompanied by surfactant dysfunction, alveolar instability, and microatelectasis. Next, denudation of basal lamina, closing of the alveoli lumen due to the proliferation of epithelial cells, and thickening of the septum occur. This suggests that early surfactant dysfunction is a key factor in the initiation of irreversible fibrotic changes and that early therapy with exogenous surfactants may restore the damaged respiratory system and prevent fibrosis [30,50]. 

Crucial factors that can contribute to high COVID-19 mortality include reduced surfactant production, its altered composition, and mutations in the genes encoding for it [8,44,50,51]. Mirastschijski et al. suggested that, when pathological conditions are observed in the lungs of patients infected with SARS-CoV-2, the admission of pulmonary surfactant could improve blood saturation, reduce pulmonary edema, and suppress the unbalanced inflammatory reaction [23,44]. 

Moreover, various reports state that PS has the ability to identify the SARS-CoV-2 spike protein and, therefore, can mediate the activation of phagocytosis in macrophages [44,52]. This evidence taken together motivated various investigators to conduct studies, including interventional clinical trials, to evaluate the clinical efficacy of PS in COVID-19 patients. 

### 2.2. Molecular Pathway of Surfactant Damage during SARS-CoV-2 Virus Infection

The investigations performed on lung biopsy cells obtained from COVID-19 patients revealed changes in many gene expression levels, including downregulation of those encoding PS proteins and responsible for surfactant metabolism. These findings encouraged further trials, which could be the basis of future surfactant therapies in SARS-CoV-2-infected individuals [8]. 

In healthy lungs, *SFTPB* and *SFTPC* gene expression is transactivated by the TTF1-CCDC59 complex and can thus effectively regulate alveolar surface tension; however, contrary to this, in the course of SARS-CoV-2 infection, the *TTF1* and *SFTPC* genes were found to be downregulated, while *SFTPB* was overexpressed in lung tissue [8,53]. Moreover, GATA6 transcription factor mediates the activation of *SFTPA* gene transcription, the protein product of which is responsible for immune and anti-inflammation processes and also alleviates alveolar surface tension. It was found that both *GATA6* and *SFTPA* genes were overexpressed in lung tissues from COVID-19 patients, while *LMCD1* (the *GATA6* antagonist) was underexpressed [8,54,55]. 

Not only genes involved in surfactant and alveolar surface tension are deregulated as a result of SARS-CoV-2 infection. Islam and Khan reported a significant decrease in the expression level of the gene pathways responsible for cholesterol biosynthesis in lung cells from patients with COVID-19. This, as a result, could reduce the accumulation of phospholipids in the lungs. Furthermore, since the phosphatidylcholine and phosphatidylglycerol are the major surfactant phospholipids, the alterations in lipid production and properties might result in nonfunctional surfactant proteins [8,56]. 

The aforementioned changes can be induced upon SARS-CoV-2 virus presence and also through excessive host response to infection. In particular, some viral proteins, e.g., NSP12 and NSP5, can indirectly modulate the SP-B and SP-C protein level, by recruiting HDAC2 and thus downregulating *TTF1* expression, which is necessary for the aforementioned surfactant protein performance. Moreover, several other viral proteins can indirectly target the SP-A and SP-D factors, which, in turn, can suppress the production of SP in the lungs, thereby deteriorating the patient condition. In addition, surfactant recycling can be disturbed upon SARS-CoV-2 infection, for example, by targeting the CSF2RA factor from the CSF2RA-CSF2RB complex, which is involved in maintaining surfactant reuptake balance. Furthermore, the reduction in *SFTPD* and *SFTPC* expression together with their regulatory partners as a result of viral factors can alter immunomodulation and efficient air exchange, leading to severe damage to lung tissue [8,53,57,58]. Table 1 presents the key molecular changes involved in surfactant dysfunction.

Overall, many reports suggest that transcription of the surfactant genes and the related protein production and metabolism are interrupted during SARS-CoV-2 lung infection and these alterations might be facilitated upon virus presence and lead to lethal complications. Additionally, in SARS-CoV-2-affected lungs, surfactant protein production and metabolism is found to be dysregulated not only by viral influence, but also by incorrect host responses [8,29].

### 2.3. Surfactant Therapy as a Potential Target in the Treatment of Acute Respiratory Distress Syndrome in COVID-19 Patients

From the abovementioned information, it appears clear that one of the major risk factors for severe COVID-19 complications is the reduction or qualitative change in pulmonary surfactant. Since the PS serves a protective and defensive role to viral infection, various researchers have suggested that administering an exogenous “natural” surfactant to COVID-19 pneumonia patients at an early stage of disease could improve lung function, shorten mechanical ventilation time, and enhance patient recovery [29,35]. 

Surfactant treatment has already been used successfully for other respiratory problems. There has been tremendous success in treating neonatal RDS with exogenous surfactant therapy, and the WHO recommends this therapy for neonatal RDS [59]. During the first waves of the pandemic, a large number of individuals with COVID-19 had symptoms of respiratory failure that qualified them for the administration of exogenous PS. 

Moreover, pulmonary surfactant proteins and therapy with surfactants in the course of SARS-CoV-2 infection is the focus of many current clinical trials. One of them, trial NCT04650191, is concerned with evaluating SP genetic variants and their connection to COVID-19 severity. Similarly, NCT04618861 is a case–control study focused on SP-D levels and their influence on infection severity, although unfortunately, the results are yet to be published. Another clinical trial (NCT04609488) is concerned with evaluating surfactant protein levels in SARS-CoV-2-infected patients and the connection between surfactant deficiency and disease progression; however, once more, the results are not yet available. 

Overall, seven clinical trials are registered concerning exogenous surfactant preparations, although no outcomes have been published to date [60]. 

The results of initial preclinical studies among COVID-19 patients on the use of surfactant preparations are partially available and have produced conflicting results; nevertheless, the effects seem promising and indicate the need for more extensive research. They draw attention to the possible early initiation of surfactant therapy and the necessity to measure the concentration of these proteins before administration [44]. Several other factors should also be considered when using an exogenous pulmonary surfactant, such as the mechanical ventilation strategy, the duration of treatment, the dose administered, the route of administration, and the formulation used [60]. 

## 3. Coagulation Processes in Pneumonia Caused by SARS-CoV-2 Infection

When the first cases of pneumonia that developed in patients with confirmed SARS-CoV-2 infection were described, disturbances in the homeostatic process were also noted. The course of the disease in the majority of patients was mild, while some developed severe acute respiratory syndrome, which required hospitalization. The disease process primarily involved the respiratory system; however, other organs were also quite often damaged to different degrees. Certain patients, specifically those with the severe course of the disease, developed hypercoagulability phenomenon associated with an unfavorable prognosis. The laboratory tests performed in patients with COVID-19, especially those who were hospitalized, confirmed numerous disorders of the hemostatic process [61]. On the basis of the available clinical data from the first waves of the pandemic, it can be estimated that approximately 14.1% of patients with COVID-19 developed venous thromboembolism (VTE), and this value increased to approximately 22.7% in those admitted to hospital [62].

### 3.1. Etiopathogenesis of Clotting Processes in Lungs among COVID-19 Patients

The vascular endothelium represents a key barrier between circulating blood and tissue. It separates platelets from the subendothelial layer, which is the main platelet activation factor and initiates clotting. The elevated levels of vascular endothelial activation markers, including von Willebrand factor (VWF), plasminogen activator inhibitor, intercellular adhesion molecule-1 (ICAM-1), and vascular cell adhesion molecule-1 (VCAM-1), have been observed in patients with COVID-19 [63]. In addition, postmortem studies confirmed damage to both endothelial cells and the connections between them in the pulmonary microvasculature [64]. In inflammatory conditions, the interaction between platelets, coagulation factors, and the vascular endothelium enhances the local and generalized inflammatory process. In the course of COVID-19, platelets become hyperactivated, which causes them to release a number of factors from their interior, activating endothelial cells and promoting the clotting process [65]. This is manifested as an altered expression of the *S100A8* and *S100A9* genes in megakaryocytes, affecting the function of platelets and also endothelial cells [66]. In the presence of platelets from COVID-19 patients, endothelial cells were shown to be activated and relaxed, resulting in increased vascular permeability. In addition, the expression profile of 1288 genes affecting intercellular junctions or inducing the inflammatory process was altered in endothelial cells in the presence of platelets from COVID-19 subjects [67]. Genes whose expressions were altered in endothelial cells under the influence of COVID-19 included *TIMP1*, *MMP2*, *MMP11*, *VWF*, *ICAM1*, and *ICAM2*, which affect coagulation and the development of inflammation. The proteins that were identified to be altering vascular endothelial cell function were MRP8 and MRP14, which are encoded by the *S100A8* and *S100A9* genes [68].

### 3.2. COVID-19 and an Alternative Way of Activating Platelets 

Excessive platelet activation is particularly noticeable in patients with a severe course of COVID-19. Comparing the levels of platelet activation markers CD62P (P-selectin) and CD63 to dense granule secretion allowed the confirmation of their significantly higher levels in the severe course of the disease in reference to the control group, and in reference to subjects with a moderate or mild course of the disease [69]. In addition, incubation of platelets from healthy subjects with plasma from severe COVID-19 patients resulted in the increased surface expression of platelet activation markers [70]. Further studies also confirmed that P-selectin is the main protein that mediates signaling to monocytes, as reflected in the increased formation of aggregates between platelets and monocytes in patients with severe COVID-19 [71]. Such aggregation enhanced the surface expression of tissue factor (TF) in monocytes, which is responsible for activating the extrinsic coagulation pathway. The observation of increased TF expression in monocytes further correlated with disease severity and was also observed in fatal cases [72]. However, in the case of COVID-19, there is also an alternative way of activating platelets, which is initiated after the spike protein binds to its receptor, ACE2, which is expressed on a number of cells, including platelets, alveolar epithelial cells, monocytes/macrophages [73]. The binding of these cells to spike proteins redirects their function towards prothrombotic and thrombogenic activities. After binding to the receptor, the amount of these proteins on the cell surface is decreased, which further reduces the level of conversion of angiotensin II to angiotensin 1 to 7. An increased angiotensin II level induces vasoconstriction, and a reduction in angiotensin 1 to 7 levels enhances the proinflammatory response and the adhesion of leukocytes to platelets [74]. The SARS-CoV-2 virus can also bind to the ACE2 receptor on the surface of platelets and megakaryocytes, which causes the release of substances accumulated in alpha granules, i.e., thrombin, VWF, PF4, adenosine diphosphate, and serotonin, which, in turn, enhance the platelet aggregation process. The situation arises as a result of increased surface expression of tissue factor on platelets following cytokine storm accompanying the infection, which activates the extrinsic coagulation pathway [75].

### 3.3. Molecular Bases of Increased Activity of Von Willebrand Factor during SARS-CoV-2 Infection

Another important factor contributing to a significantly increased risk of thrombosis in patients with COVID-19 is high activity of VWF, which is released from the alpha granularity content during degranulation of previously activated platelets or from endothelial cells. The factor is synthesized in the form of ultralarge particles and is responsible for the adhesion of platelets to the subendothelial layer. The activity of VWF is influenced by the ADAMTS13 protein (a disintegrin and metalloproteinase with a thrombospondin type 1 motif, member 13), which has the ability to split ultralarge molecules into smaller fragments that are biologically active. However, failure to split ultralarge VWF molecules will be associated with an increased risk of intravascular coagulation [76]. The release of VWF itself is initiated by the activation of platelets or endothelial cells. In the course of COVID-19, ADAMTS13 activity decreases, which is associated with an increase in VWF activity and a higher thrombotic risk. Moreover, the increase in the activity of VWF correlates with the severity of the disease, which indirectly reflects the degree of activation of platelets. VWF also plays a role in secondary hemostasis, i.e., it protects coagulation factor VIII against proteolysis via active protein C. In the course of COVID-19, factor VIII activity also intensifies, becoming another component of the hypercoagulable state [77].

### 3.4. Molecular Abnormalities Cause Hypercoagulability States in Patients with COVID-19 

Hypercoagulability induced by overexpression of TF on endothelial cells during SARS-CoV-2 infection may also be due to the activation of Toll-like receptors (TLRs). TLRs are expressed on various cells of innate immunity, including monocytes, macrophages, and dendritic cells in the alveolar microenvironment. Pulmonary macrophages and those that have entered the circulation complete immunological supervision in the lungs. When the virus enters the respiratory system, epithelial and endothelial cells in the blood vessels in the lungs become infected. The genetic material of the virus causes the activation of TLRs on these cells, but also the remnants of already disrupted cells cause their stimulation. TLR3 can be activated by single-stranded virus RNA such as SARS-CoV-2 and in silico analyses confirmed that TLR7 and TLR8 possess the same property. Moreover, in silico studies also confirmed that TLR4 is responsible for molecular recognition of spike proteins. Activation of this receptor causes interferon release, which has protective properties in the case of viral infections. However, it has been observed that activation of this receptor by the viral genetic material leads to surface overexpression of the tissue factor on endothelial cells and its activation enables the initiation of the extrinsic coagulation cascade [78]. 

Coagulation proteins are also committed to the occurrence of the hypercoagulability phenomenon in the course of COVID-19 and are directly related with the virus particle. Cytoplasmic poly (A) 4 binding protein (PABPC4) interacts with the nucleocapsid (N) protein. In addition, the vitamin K epoxy reductase complex subunit 1 (VKORC1) interact with the SARS ORF7a protein. The PABPC4 protein is found on the surface of thrombin-activated platelets and plays an important role in regulating the translation of polyadenylate-stabilizing proteins in platelets and megakaryocytes. Another protein that influences the viral proteins is VKORC, which is a key enzyme associated with vitamin K turnover. Vitamin K, among others, is responsible for the post-translational carboxylation of glutamine residues of certain coagulation factors (II, VII, IX and X), enabling them to become active on the negatively charged surface of phospholipids. The interaction between viral and human proteins may alter their function or their affinity for other structures. SARS-CoV-2 infection affects the level of carboxylation of some coagulation factors, leading to the hypercoagulability phenomenon [79].

### 3.5. Molecular Alterations of the Classical and Lectin Complement Pathways Affecting the Process of Hemostasis 

SARS-CoV-2 infection is responsible for the activation of the classical and lectin complement pathways, thus affecting the process of hemostasis. In the case of COVID-19, the classical complement pathway can be activated after the virus has been present in the body for a week via IgG/IgM-SARS-CoV-2 immune complexes [80]. However, the lectin pathway is activated earlier, upon exposure to the virus, by the binding of mannose-binding lectin (MBL), which is associated with homodimers of MBL-associated serine proteases (MASPs), to viral N-Glycan or immune complexes formed by IgA immunoglobulin and SARS-CoV-2. The MASP of the lectin activates platelets and TF causing the hypercoagulability phenomenon. The complement lectin pathway is a part of an innate response, triggered by pattern recognition molecules (PRMs), including two collectins (MBL and collectin-LK) and three ficolins (ficolin-1, -2, -3) that initiate complement activation upon binding to carbohydrates present on the surface of microbes or altered tissues [81]. *MASP1* and *MASP2* genetic polymorphisms, which are associated with increased or decreased protein concentration, modulate the activation of the complement lectin pathway in response to various types of diseases. MASP proteins play a crucial role in the interaction between the complement system, coagulation, and fibrinolysis cascades, generating a proinflammatory and prothrombotic response [82]. MASP-1 substrates are not only limited to components of the complement system, but also include various members of the coagulation system, including factor XIII (FXIII), quininogen, protease-activated receptor 4 (PAR4) in endothelial cells, prothrombin, and the thrombin-activating fibrinolysis inhibiting antifibrinolytic factor (TAFI). Increased activation of the coagulation system in the initial phase of COVID-19, which manifests as a higher concentration of D-dimers, a decreased number of platelets, and slightly prolonged prothrombin time, seems to be caused by the interaction between MASP-1, MASP-2, and thrombin, which could explain the coexistence of DIC syndrome in the early stages of COVID-19. MASP-1 and MASP-2 exhibit a catalytic role against prothrombin and fibrinogen, activating the coagulation system and providing an explanation for the pathogenesis of diseases in which the activation of the coagulation system occurs in response to inflammation. Low-molecular weight heparin (LMWH) and antithrombin therapies appear to have a positive effect as a prevention against early stage DIC in COVID-19. LMWH and antithrombin are also inhibitors of MASP-1, as they block the lectin pathway of complement activation and the coagulation cascade [83]. Table 2 summarizes the processes connected to coagulation in the lungs of COVID-19 patients.

### 3.6. The Coexistence of Coagulation Processes and Changes in the Fibrinolysis Process in the Course of SARS-CoV-2 Infection

One of the complications in the course of SARS-CoV-2 is disorder of the coagulation process and the coexistence of changes in the fibrinolysis process. Disturbances in fibrinolysis are an unfavorable prognostic factor and more common in patients with severe disease, increasing mortality among them. However, these changes differ from those occurring in DIC syndrome, e.g., as a result of sepsis, since COVID-19 usually shows elevated D-dimer values with normal clotting times. The increased levels of D-dimers in COVID-19 patients not only indicate that the disease causes a strong activation of the coagulation system, but also abnormalities in fibrinolysis. The basic difference between hemostasis disorders in COVID-19 and DIC is the almost complete inhibition of the fibrinolysis process despite the increased D-dimer values [84,85]. In the course of COVID-19, strong activation of the coagulation cascade leads to the formation of large amounts of fibrin polymers, but without the activation of fibrinolysis. This can cause the formation of huge amounts of fibrin degradation products, D-dimers, which are associated with initial transient hyperfibrinolysis. These fibrinolytic disorders can result in a longer fibrin degradation process and lead to thromboembolic complications. The mechanism of these abnormalities is not fully understood, and its elucidation would allow us to predict the potential causes of these disorders and to modify therapeutic activities [85,86]. 

Changes in fibrinolysis may be associated with damage to the epithelial and vascular endothelial cells in the lungs by the SARS-CoV-2 virus and the release of tissue plasminogen activator. The most important inhibitor of the fibrinolysis process, which inhibits the activity of both t-PA (encoded by the *PLAT* gene) and u-PA (encoded by *PLAU* gene), is the plasminogen activator 1 inhibitor (PAI-1), which is encoded by the *SERPINE1* gene. Plasminogen activators are responsible for the transformation of inactive plasminogen into enzymatically active plasmin, which is based on the cleavage of fibrin and the formation of its degradation products, including D-dimers. The available studies indicate that alveolar epithelial cells in the course of SARS-CoV-2 virus infection exhibit lower expression levels of the genes encoding uPA and its receptor, while the *PLAT* and *PLG* (encoding plasminogen) genes exhibited higher levels of expression. These changes in correlation with the increased level of *FGB* gene transcripts disrupt local fibrinolysis and predispose to pulmonary thrombosis [87,88,89]. 

Dysfunction of the coagulation and fibrinolysis processes in COVID-19 is associated with acute inflammatory process and cytokine storm. An excessive immune response to SARS-CoV-2 infection is the fundamental cause of hemostatic dysfunction, in which the predisposition to thrombosis is predominant. One of the most common and more severe complications of COVID-19 is the development of ARDS, which is accompanied by hypercoagulability and thrombus formation in the pulmonary microcirculation. The phenomenon most commonly associated with acute lung injury in ARDS is the suppression of the fibrinolytic system [90]. The inducer of the secretion of large amounts of PAI-1 may be the inflammatory process and the accompanying secretion of proinflammatory cytokines. In COVID-19, inhibition of plasmin production by PAI-1 is not dominant as compared to other diseases with impaired hemostasis, such as pneumonia. In patients who develop this disorder with SARS-CoV-2 infection, fibrinolytics such as heparin appear to be introduced to prevent the development of pulmonary thromboembolism, which is a severe complication. Another therapeutic approach that may have benefits in patients with severe COVID-19 pneumonia is the use of exogenous t-PA or plasminogen directly. A reduction in respiratory failure in COVID-19 patients with plasminogen treatment may suggest large deficits in endogenous plasminogen levels in these patients [88,90,91,92]. 

Another pathway leading to the inhibition of fibrinolysis is the decreased activity of TFPI, i.e., an inhibitor of the tissue factor-dependent pathway. TFPI is responsible for endogenously inhibiting coagulation in the step of blocking the activation of factor VII, preventing the activation of factor X, and the subsequent coagulation cascade. In the infection, the coagulation cascade is strongly activated by the extrinsic pathway, with the participation of the tissue factor, due to its release from the damaged vascular endothelium. Maintaining the balance between the activity of TF and TFPI is important in terms of the occurrence of thrombotic complications. In COVID-19 infection, this balance is disturbed. The infection leads to an increase in TF protein activity, without affecting the expression of TFP1, which contributes to the intensification of thrombotic processes [78,93]. 

The anticoagulant proteins involved in the pathogenesis of coagulation disorders in the course of COVID-19 include thrombomodulin (encoded by the *THBD* gene) and protein C (encoded by the *PROC* gene), which are responsible for inhibiting thrombin generation at various stages of its formation. Thrombomodulin enables thrombin binding and the activation of protein C, which, together with cofactor protein S (encoded by the *PROS1* gene), inactivate the coagulation cascade by inhibiting the activation of factors V and VIII. It has been observed that severely ill patients had a higher risk of coagulopathy and increased amounts of factor V. Changes in COVID-19 include changes in the *PROS1* and *THBD* genes, the expressions of which are reduced in the course of infection, which also adversely affects the formation of thrombotic complications [78,90,94]. The introduction of anticoagulant therapy with the use of drugs such as warfarin, which affects the S protein in patients with severe COVID-19, may have great health benefits and contribute to the minimization of thrombotic events [93,95].

## 4. Pulmonary Fibrosis in the Course of SARS-CoV-2 Virus Infection

Another serious complication in patients with a history of SARS-CoV-2 infection, especially during first waves of the pandemic, is the process of pulmonary fibrosis (PF). It is a phenomenon of pulmonary parenchyma remodeling, in which impaired regeneration of damaged alveoli and excessive accumulation of extracellular matrix are observed, resulting in irreversible hardening and scarring of the lung tissue [13,96]. Changes in the structure of the pulmonary parenchyma impairs gas exchange, which manifests as dyspnea, chest pain, dry cough, and fatigue [13,97]. 

Preliminary studies indicate that the PF phenomenon mainly affects patients who have developed acute respiratory failure, but the exact percentage of patients with such changes due to SARS-CoV-2 infection is not fully known [98]. Approximately 3–7% of all COVID-19 patients developed ARDS (more than 30% of whom were hospitalized) during the first waves of the pandemic [98,99]. According to certain sources, the number of patients with fibrotic changes may account for up to 44% of those hospitalized due to SARS-CoV-2 infection, making PF a serious medical problem connected with the early waves of the COVID-19 pandemic [100]. Studies have shown that there are numerous risk factors that predispose to the development of pulmonary tissue fibrosis, the most important of which is mechanical ventilation that contributes to the development of fibrosis in the lungs by direct damage to the tissue [101]. Another such factor is advanced age, which was also shown to be significant in MERS and SARS-CoV-1 infections [102]. Comorbidities are also a factor that increases the risk of developing complications in the form of fibrosis. Diseases that may affect the occurrence of complications after infection with SARS-CoV-2 include asthma and diabetes [103]. Other factors and comorbidities have been shown to affect the severity of SARS-CoV-2 virus infection, but their involvement in the development of pulmonary fibrosis has not been confirmed, including male gender, active smoking, alcohol abuse, obesity, and chronic pulmonary disease [98,101,103]. 

### 4.1. Etiopathogenesis of Pulmonary Fibrosis among COVID-19 Patients

As a result of bacterial or viral infection, the epithelium of the respiratory tract may be damaged, and this causes inflammation and initiates regeneration processes, which may end with the complete regeneration or the appearance of local fibrosis, thus limiting the organs’ functionality [104]. Negative changes in the lung parenchyma stimulate the release of inflammatory mediators and the migration of platelets, which, as a result of activation and degranulation, lead to the formation of a clot that is responsible for local vasodilation. In addition, the action of matrix metalloproteinases (MMPs) also increases the availability of damage sites for inflammatory cells, such as neutrophils, eosinophils, lymphocytes, and macrophages, by remodeling the basement membrane components [105]. Pulmonary macrophages play a very important role in this process, via removing the debris of damaged alveoli and the production of cytokines and growth factors that initiate repair mechanisms, including angiogenesis, fibroblast activation, and collagen deposition [104,106]. These processes are sufficient to repair tissue lesions when the basement membrane is intact. However, in the case of severe damage or the prolonged presence of the inflammatory factor, the activity of fibroblasts is maintained, transforming normal lung tissue into fibroblastic tissue. As a consequence, local or generalized rearrangement of the alveolar structure is observed [98,104]. The exact mechanism of PF development in COVID-19 patients is not fully understood. There are two popular hypotheses that underline which factors influence the remodeling of lung tissue [98]. The first is based on the ACE2-related profibrotic pathway being activated by SARS-CoV-2 infection. The second is related to a hyperinflammatory reaction due to the infection inducing PF.

### 4.2. ACE2-related Profibrotic Pathway Activated by SARS-CoV-2 Infection

The renin-angiotensin system (RAS) controls the volume of circulating blood and the concentration of sodium and potassium in body fluids. The key negative regulator of this system is ACE2 [107]. ACE2 is part of the ACE2/Ang- (1-7)/MasR axis, and is responsible for the hydrolysis of angiotensin II (Ang II) to Ang1-7, which directly influences the activation of the MasR receptor. The activation of this axis has anti-inflammatory and antifibrotic effects. In addition, ACE2 is also involved in the transport of amino acids and acts as receptor for the SARS-CoV-2 virus [107,108,109]. 

SARS-CoV-2 infection begins with the connection of the virus molecule to the ACE2 receptor on the membrane of type II pneumocytes, reducing ACE2 expression and ACE2/Ang-(1-7)/MasR axis activity [108]. This, in turn, results in increased levels of Ang II, which promotes inflammation and the fibrosis process. Increased plasma levels of Ang II in patients with SARS-CoV-2 were confirmed in studies by Wu et al., wherein a positive correlation between the concentration of this peptide and the severity of symptoms was demonstrated [110]. 

As a result of the decreased amount of Ang-(1,7) peptide, the concentration of transforming growth factor β (TGF-β) increases, which, through the modulation of the activity and the proliferation of fibroblasts, plays an important role in the process of fibrosis [111,112]. Activated TGF-β promotes the formation of myofibroblasts from fibroblasts and collagen synthesis, thereby regulating the amount of extracellular matrix (ECM) produced [113]. TGF-β, as one of the main profibrotic stimuli, is also directly amplified by the SARS-CoV-1 nucleocapsid protein. Since the nucleocapsid proteins of SARS-CoV-2 are 90% similar to those of SARS-CoV-1, it can be hypothesized that this is one of the possible mechanisms involved in enhancing the process of pulmonary fibrosis observed in the first waves of the pandemic [114]. 

### 4.3. Hyperinflammatory Reaction due to Infection can Induce Pulmonary Fibrosis

The release of proinflammatory cytokines is a physiological process that accompanies the entry of an infectious agent into the body. In the case of infection caused by SARS-CoV-2, the immune system is often hyperactivated, which leads to the induction of a cytokine storm. As a result of the entry of the virus into the epithelial cells of the respiratory tract, there is strong activation of T helper 1 cells (Th1), which stimulate the intensive production of proinflammatory cytokines, such as granulocyte-macrophage colony-stimulating factor (GM-CSF) and interleukin-6 (IL-6). Monocytes, as a consequence of GM-CSF stimuli, produce a high concentration of tumor necrosis factor-α (TNF-α) and other cytokines (mainly IL-6), which is known as a cytokine storm [115]. 

A cytokine storm can induce cellular lesions of airway epithelial and endothelial cells, severe lymphopenia, neutrophils recruitment, pulmonary cell infiltration, and finally, it can lead to lung tissue injury, ARDS, and even the patient’s death [116]. If this phenomenon is not suppressed, the progressive damage and regeneration processes can result in remodeling of the lung tissue and development of fibrosis [98]. 

A large number of scientific papers indicate that the severity and mortality of COVID-19 observed during early pandemic stages are associated with high proinflammatory cytokine levels in serum [115,116]. Studies by Xiong et al. showed that the expression level of the *IL-6* gene, measured in peripheral blood samples, did not significantly differ between a patient with COVID-19 and the control group. This indicates that the elevated IL-6 values present in the serum of patients with COVID-19 are probably secreted by lung epithelial cells [117]. Results published by Diao et al. indicate that patients with severe COVID-19 have very low levels of CD4+ and CD8+ T cells. These values were inversely proportional to the concentration of proinflammatory cytokines, such as IL-6, IL-10, and TNF-α. It is worth noting that during the recovery period, T cell values increased and proinflammatory cytokine levels decreased significantly [118]. Moreover, IL-1β, which is one of the most important proinflammatory cytokines with pleiotropic properties, is significantly elevated in the serum of COVID-19 patients as compared to healthy people. In addition, patients with COVID-19 have a higher expression of the gene encoding this interleukin in peripheral blood cells than healthy individuals [116]. A study conducted by Colarusso et al. showed characteristic changes in the cytokine profile that may predispose to pulmonary fibrosis. Post-COVID patients who developed fibrotic changes had higher levels of IL-1α and TGF-β, but lower values of IFN-β as compared to patients who also had COVID-19, but did not develop lung fibrosis [119]. 

### 4.4. Potential Molecular Markers of Pulmonary Fibrosis in the Course of SARS-CoV-2 Virus Infection

Serum biomarkers of PF have been classified according to the mechanism responsible for fibroproliferation. They include the following: alveoli damage, including the Krebs von den Lungen antigen (KL-6); fibrogenesis and extracellular remodeling, including different matrix metalloproteinase (e.g., MMP3, MMP7, MMP9); growth factors and adhesion molecules, including TGF-β, vascular endothelial growth factor (VEGF), insulin-like growth factor (IGF), chitinase-3-like protein 1 (YKL-40); and chemokines, including IL-6 and IL-8. However, despite many previous and ongoing studies, detailed recommendations for the use of PF markers in patients following COVID-19 have not yet been established [120]. 

KL-6 is a glycoprotein that is mainly expressed on the surface of damaged type II alveolar cells. It is used as a marker of various types of lung tissue damage, including the process of fibrosis. A retrospective study by Peng et al. carried out in 2020 showed that higher KL-6 serum concentrations were observed in patients with severe COVID-19 with symptoms of PF than in the group without PF [121]. A study conducted by Arnold et al. did not show any correlation between the serum concentration of the KL-6 antigen and the severity of disease symptoms at patient admission to hospital and after 28 days of hospitalization. However, patients with an abnormal lung tissue morphology assessed at 12 weeks postinfection had significantly higher levels of the KL-6 antigen as compared to the group whose lung morphology was normal [122]. The use of the KL-6 antigen as a predictive marker for the development of pulmonary fibrosis following COVID-19 has also been proposed by Xue et al. The study showed statistically higher values of KL-6 in the serum of patients who developed PF. It was also demonstrated that the KL-6 serum values were significantly higher in patients with irreversible fibrosis as compared to those with reversible fibrosis [123]. Summarizing the results of the presented work and the meta-analysis carried out by Naderi et al., the KL-6 antigen may have predictive and prognostic application in patients with COVID-19. This is probably due to the fact that damage to alveolar epithelial cells leads to the production of a large amount of the KL-6 antigen, which in several mechanisms, including interaction with the TGF-β signaling pathway, may promote the process of PF [124]. 

YKL-40 is a small protein secreted by neutrophils, macrophages, chondrocytes, and cancer cells. It probably plays an important role in the process of cell profiling and differentiation, inflammation, and remodeling of the extracellular matrix. It is also involved in an alternative mode of macrophage activation [120]. A systematic review conducted by Tong et al. showed that YKL-40 serum levels were higher in patients with interstitial lung disease (ILD), which includes PF, than in the control group [125]. Similar results were also obtained by Majewski et al. in which the YKL-40 serum concentration was statistically significantly higher in patients with idiopathic pulmonary fibrosis than in healthy people [126]. Early positive results of YKL-40 protein research, obtained in various lung diseases, have become the basis for the analysis of this parameter in patients with COVID-19. In COVID-19 patients, the YKL-40 concentration is significantly higher than in healthy subjects, but is also significantly higher than in patients with obstructive pulmonary disease (COPD) and ILD. In the same study, YKL-40 was considered as an indicator of COVID-19 severity, as significantly higher concentrations of this protein were observed in patients who required hospitalization in the intensive care unit compared to patients with a milder course of disease during first waves of the pandemic [127]. In a study by Kimura et al., high YKL-40 values were also associated with the severe course of COVID-19, but also with an unfavorable prognosis [128]. On the basis of the presented results, it can be suggested that YKL-40 plays an important role in the pathogenesis of COVID-19, especially as regards lung tissue remodeling. 

Another frequently proposed and studied marker that can be used in COVID-19 is the family of extracellular endopeptidases known as MMPs. MMPs are a group of zinc-dependent proteolytic enzymes the main function of which is remodeling of the extracellular matrix. In addition, these proteins mediate processes such as wound healing, angiogenesis, immunity, cell migration, proliferation, signaling pathways, leukocyte activation, and more. Under physiological conditions, MMPs occur in small amounts in tissues; however, increased secretion is observed in various diseases, such as in cancer, cardiovascular diseases, and in acute or chronic lung diseases [129,130]. In a study conducted on 53 patients with a severe COVID-19 course, increased MMP-9 values and decreased MMP-2 values in their plasma were observed compared to the control group. The correlation between MMP-2 values and survival was also assessed, wherein higher values of this metalloproteinase were observed in patients who died as a result of infection compared to those who survived. For MMP-9, the authors showed no such association [131]. Another study showed a negative correlation between the concentration of MMP-9 in patient serum and their clinical stage. A significant decrease in the P/F ratio (an indicator used to assess the severity of hypoxemia and trend progression of respiratory failure) was observed when the plasma concentration of MMP-9 was increased [132]. Chavez et al. reported significantly higher concentrations of MMP-7 in patients with severe COVID-19 and proposed MMP-7 as a useful marker for predicting the use invasive mechanical ventilation. It was also noted that high levels of MMP-7 were maintained for several months in patients who developed fibrotic changes in the lungs [133]. Higher values of MMP-7 were also observed in another study in patients who had fibrotic lesions detected in the CT scan compared to patients who did not develop such lesions after being diagnosed with COVID-19 during the early stages of the pandemic [1]. The presented studies showed that metalloproteinases might have potential utility in the prognostic assessment of the severity of COVID-19 (including a role as an early indicator of respiratory failure) and the assessment of the probability of long-term complications in the form of fibrotic changes. Table 3 summarizes the changes observed in the COVID-19 lungs involved in fibrosis processes.

## 5. Conclusions

The three previously described pathways are severely disrupted after SARS-CoV-2 entry and were responsible for the difficulties in managing patient symptoms during the early stages of the pandemic, leading to a vicious cycle. The observed changes and dysregulation also contribute to increased mortality. This is why it is crucial to develop a therapeutic strategy that includes the considerations presented in this study for the effective management of patients with COVID-19. 

There is an urgent need to understand both the molecular and biochemical bases of the observed changes and to systematize the knowledge already acquired. This will enable medical professionals to establish of an individualized approach and to tailor therapies based on changes observed in the lung physiology and morphology via imaging. Moreover, this will aid in the identification of biological subtypes and phenotypes of infection, and, as a consequence, improve the treatment outcomes for COVID-19 and its possible future variants. 

This, in turn, will help to stratify the risk of serious complications in patients and to select those who may develop complications before a clinical manifestation has been observed.

## Figures and Tables

**Table 1:** Processes in the COVID-19 lungs related to surfactant dysfunction.

Surfactant		
Upregulation	Downregulation	SARS-CoV-2 Infection Effects
-	*TTF1* *SFTPC* *SFTPD*	Altered immunomodulation Altered air exchange Ineffective alveolar surface tension regulation
*SFTPB*	-	Ineffective alveolar surface tension regulation
*GATA6* *SFTPA*	-	Disrupted immune and anti-inflammation processes
-	*LMCD1*	Disrupted immune and anti-inflammation processes
*SREBP2*	cholesterol biosynthesis genes	Reduction in the phospholipids in the lungs Nonfunctional surfactant proteins Cytokine storm induction

**Table 2:** Processes connected to coagulation in the lungs of COVID-19 patients.

Coagulation		
Upregulation	Downregulation	SARS-CoV-2 Infection Effects
Von Willebrand factor, ICAM-1, VCAM-1	-	Platelets activation Clotting initiation Increased risk of thrombosis
*TIMP1, MMP2, MMP11, VWF*	-	Impacted coagulation processes - Platelet adhesion Development of inflammation
CD62P (P-selectin) TF (tissue factor)	-	Excessive platelet activation Initiation of the extrinsic coagulation cascade Induction of hypercoagulability
PABPC4, VKORC1	ADAMTS13	Induction of hypercoagulability
TLR3, TLR4, TLR7, TLR8	-	Induction of hypercoagulability Initiation of the extrinsic coagulation cascade
MASP1, MASP2	-	Activation of the classical and lectin complement pathways Catalytic role against prothrombin and fibrinogen
*SERPINE1*	-	Inhibition of the fibrinolysis process
*PLAT* *PLG* *FGB*	uPA uPAR	Local fibrinolysis reduction and pulmonary thrombosis predisposition D-dimer production
-	*THBD, PROS1*	Development of thrombotic complications

**Table 3:** Processes in the COVID-19 lungs involved in fibrosis formation.

Fibrosis		
Upregulation	Downregulation	SARS-CoV-2 Infection Effects
TGF	ACE2/Ang- (1-7)/MasR axis	Inflammation and fibrosis promotion
Th1 cells, GM, CSF, IL-6, IL-10, IL 1, IL-1, TNF	-	Cytokine storm induction Remodeling of the lung tissue/fibrosis
KL-6 antigen	-	Pulmonary fibrosis promotion
*MMP9, MMP7*	*MMP2*	Pulmonary fibrosis promotion
YKL-40	-	Important role in the tissue remodeling

## Data Availability

Not applicable.
